# Value-based decision-making predicts alcohol use and related problems in young men

**DOI:** 10.1177/02698811231212151

**Published:** 2023-11-23

**Authors:** Johannes Petzold, Angela Hentschel, Hao Chen, Sören Kuitunen-Paul, Edythe D London, Andreas Heinz, Michael N Smolka

**Affiliations:** 1 Department of Psychiatry and Psychotherapy, Technische Universität Dresden, Dresden, Germany; 2 Institute of Clinical Psychology and Psychotherapy, and Department of Child and Adolescent Psychiatry, Technische Universität Dresden, Dresden, Germany; 3 Chair of Clinical Psychology and Psychotherapy, Technische Universität Chemnitz, Chemnitz, Germany; 4 Department of Psychiatry and Biobehavioral Sciences, Department of Molecular and Medical Pharmacology, and the Brain Research Institute, University of California at Los Angeles, Los Angeles, CA, USA; 5 Department of Psychiatry and Neurosciences at Charité Campus Mitte, Charité Universitätsmedizin Berlin, Berlin, Germany

**Keywords:** Delay discounting, probability discounting, loss aversion, alcohol use disorder, binge drinking

## Abstract

**Background:**

Alcohol consumption is a leading cause of morbidity and mortality worldwide, disproportionately affecting young men. Heavy episodic drinking is particularly prevalent among men, with this behavior peaking between the ages of 20 and 24.

**Aims:**

We sought to identify dimensions of decision-making in men that would predict the development of hazardous alcohol use through emerging adulthood.

**Methods:**

This prospective observational study profiled value-based decision-making in 198 healthy men at age 18 and assessed their alcohol involvement annually until age 24. Latent growth curve modeling estimated individual variability in trajectories of alcohol involvement and regressed this variability on five choice dimensions.

**Results:**

Low loss aversion predicted sustained heavy episodic drinking from age 18 to 24. Both high delay discounting and risk-seeking for gains independently predicted a considerably higher cumulative alcohol use during these 6 years, with high delay discounting indicating escalating consumption from age 21. Risk-seeking for gains additionally predicted meeting more criteria for Alcohol Use Disorder in these 6 years. Risk-seeking for losses was not significantly related to alcohol outcomes. Choice preferences were largely independent of each other but were correlated with choice consistency, with low consistency predicting heavy episodic drinking from age 18 to 24 beyond these associations.

**Conclusions:**

The predictive effects collectively suggest that overvaluing immediate and probabilistic incentives, rather than underestimating harm, drives hazardous drinking in young men. The differential relations of choice preferences and consistency to alcohol involvement through emerging adulthood provide distinct cognitive-behavioral patterns that warrant consideration in the development of harm reduction interventions.

## Introduction

### Alcohol use problems in young men

Alcohol use is a leading cause of morbidity and mortality worldwide, disproportionately affecting young men (Bryazka et al., 2022; World Health Organization, 2018). The global prevalence of heavy episodic drinking is particularly high in men and peaks between the ages of 20 and 24 (World Health Organization, 2018), highlighting the importance of emerging adulthood as a period when drinking patterns and related problems develop, resolve, or escalate (Chassin et al., 2013; Thompson et al., 2014; Wichers et al., 2013; Zucker, 2015). These trajectories are shaped by numerous factors, such as genetic variations, personality traits, social networks, and environmental stimuli (Chassin et al., 2013; Hinckers et al., 2006; Wichers et al., 2013; Zucker, 2015).

### Goal-directed behavior and impulsive decision-making

Internal and external factors influence drinking by ultimately affecting decisions regarding alcohol use, based on the assignment of subjective values to states, goals, and actions (Chassin et al., 2013; Goschke, 2014; Guttman et al., 2018). Goal-directed behavior adapts to new experiences, contexts, and demands (Goschke, 2014). This requires cognitive control to capture, maintain, and exploit relevant information while suppressing distracting stimuli, unwanted thoughts, and prepotent responses (Goschke, 2014). Low cognitive control causes inconsistent and impulsive choices by limiting attention, patience, foresight, planning, and reflection (Goschke, 2014). These largely independent impulsive expressions promote drinking, which is reinforced by immediate rewards (e.g., stimulation, relaxation) despite delayed costs (e.g., intoxication, withdrawal) and various risks (e.g., accidents, addiction; Camchong et al., 2014; Herman and Duka, 2020).

### Assessment of value-based decision-making

Paradigms comprising a series of financial offers can measure trade-offs among varying amounts, delays, and probabilities (Pooseh et al., 2018). Delay discounting (V = A / (1 + k D)) and probability discounting (V = A / (1 + k (1 − p) / p)) quantify the degree of hyperbolic decline (k) in subjective values (V) of offered amounts (A) with increasing delay (D) and decreasing probability (p), respectively. People generally exhibit risk aversion in probability discounting for gains, as they prefer smaller, certain gains to larger, uncertain gains (Kahneman and Tversky, 1984). By contrast, they exhibit risk-seeking in probability discounting for losses by accepting risks of higher losses rather than taking certain losses (Kahneman and Tversky, 1984). In mixed gambles (V = ½ (G − λ L)), they exhibit loss aversion (λ) by being reluctant to gamble with a 50–50 chance if possible gains (G) are not substantially greater than possible losses (L; Kahneman and Tversky, 1984).

### Decision-making and alcohol outcomes

People with moderate or severe Alcohol Use Disorder tend to favor smaller, sooner over larger, later rewards, and this preference seems to be associated with earlier disorder onset, heavier drinking, and greater disorder severity (Camchong et al., 2014; Herman and Duka, 2020). Chronic alcohol use may also be linked to low loss aversion (Bernhardt et al., 2017), possibly independent of strong delay discounting (Thrailkill et al., 2022). Conflicting reports on delay discounting and loss aversion have appeared, especially concerning non-dependent drinking (Campbell et al., 2021; Herman and Duka, 2020; Mayhew et al., 2020; Poulton et al., 2022; Stancato et al., 2020; Tucker et al., 2021; Zorick et al., 2022). Delay discounting cannot reliably distinguish between different levels, patterns, or problems of non-dependent drinking (Campbell et al., 2021; Herman and Duka, 2020; Mayhew et al., 2020; Poulton et al., 2022; Stancato et al., 2020; Tucker et al., 2021). Similarly, the scarce research on probability discounting indicates that risk-seeking for gains and risk aversion for losses are associated with moderate to severe Alcohol Use Disorder but possibly not with less problematic drinking (Bernhardt et al., 2017). Furthermore, alcohol has no general acute effects on delay or probability discounting or loss aversion (Bernhardt et al., 2019; Herman and Duka, 2019). These findings together suggest that delay discounting, risk-seeking for gains, and risk aversion for losses increase and loss aversion decreases after the transition to Alcohol Use Disorder. Notably, risk aversion for losses, but not delay discounting, risk-seeking for gains, or loss aversion, predicted relapse to heavy drinking within a year in recently abstinent patients with Alcohol Use Disorder (Bernhardt et al., 2017).

Discrepancies in value-based decision-making among people who use alcohol without meeting criteria for moderate or severe Alcohol Use Disorder reflect the heterogeneity in definitions (e.g., threshold for low-risk consumption), measures (e.g., frequency, quantity), and periods (e.g., past month or year) of drinking (Kalinowski and Humphreys, 2016; Thompson et al., 2014), including dichotomizations (e.g., non-binging vs. binging). Findings might also be confounded by gender effects, considering gender differences in decision-making (Green and Myerson, 2019) and alcohol involvement (World Health Organization, 2018). Moreover, drinking patterns develop differentially across the lifespan (Chassin et al., 2013; Thompson et al., 2014; Wichers et al., 2013; Zucker, 2015) and may thus relate differentially to dimensions of decision-making. Cross-sectional studies on one choice aspect cannot delineate predictive or independent effects on drinking, which may stunt the cortical maturation into young adulthood that underlies increasing cognitive control and choice consistency as well as decreasing delay discounting, risk-taking, and loss aversion (Camchong et al., 2014; Guttman et al., 2021; Ripke et al., 2012; Zucker, 2015).

### Project overview, results, and hypotheses

The bicentric project Learning and Habituation as Predictors of the Development and Maintenance of Alcoholism (https://gepris.dfg.de/gepris/projekt/186318919?language=en) included our studies on value-based decision-making in young men (ClinicalTrials.gov Identifiers: NCT01744834, NCT01858818). A blood alcohol concentration of 0.08% did not change delay discounting, probability discounting for gains, for losses, or loss aversion in 18-year-old men (Bernhardt et al., 2019). Stronger delay discounting but not probability discounting for gains, for losses, or loss aversion at age 18 was associated with higher chronic alcohol use, and none of these dimensions predicted changes in alcohol consumption at age 19 (Bernhardt et al., 2017). Yet, risk-seeking for losses at age 18 indicated greater functional connectivity of the default mode and left frontoparietal networks to their adjacent areas relevant for decision-making and future thinking (Deza Araujo et al., 2018). Effects of alcohol involvement on changes in decision-making between the ages of 18 and 21 are currently being investigated.

The present work modeled choice dimensions simultaneously to predict dimensional measures of alcohol outcomes longitudinally over 6 years. Risk-seeking for gains, risk aversion for losses, low loss aversion, and high delay discounting at age 18 were hypothesized to confer differential risks for peak alcohol involvement between the ages of 18 and 24. Differential relationships were expected to shape distinct curvilinear trajectories, mapping these decision tendencies on higher cumulative consumption, longer intervals of heavy episodic drinking, and steeper progression to Alcohol Use Disorder. Such cognitive-behavioral patterns would provide mechanisms for advancing risk stratification and targeted interventions in young men.

## Methods

### Participants and procedures

This prospective observational study was approved by the ethics committees of the Charité Universitätsmedizin Berlin and Technische Universität Dresden. All participants gave informed consent in writing and received monetary compensation.

Participants were recruited from random population samples of 18-year-old men, who were identified by resident registration offices in the German cities of Dresden and Berlin. Exclusion criteria were: <2 drinking days during the 3 months before enrollment, current or past major neurological disorder (e.g., brain trauma, multiple sclerosis) or mental disorder (other than Tobacco Use Disorder or mild Alcohol Use Disorder), use of any substance that would interfere with test performance, visual impairment despite correction, any conditions incompatible with magnetic resonance imaging, and left-handedness.

Data on value-based decision-making were available from 198 of the 201 participants who attended the baseline visit. Alcohol Use Disorder criteria, consumption per typical occasion, and average daily drinking were assessed annually over 6 years using the Composite International Diagnostic Interview (Jacobi et al., 2013). Follow-up assessments were conducted via phone, email, and websites, except for the 36-month follow-up that resembled the in-person baseline visit.

### Value-based decision-making

Delay discounting, risk aversion for gains, risk-seeking for losses, and loss aversion were each quantified by one task of a computerized test battery (available at https://github.com/spooseh/VBDM; Pooseh et al., 2018). The delay discounting task assessed preferences for smaller, immediate rewards versus larger ones available after 3, 7, 14, 31, 61, 180, or 365 days (e.g., €7 now or €10 in 7 days). Strong discounting of delayed rewards produces high values of k, the index of devaluation. The probability discounting for gains task provided choices between smaller, certain and larger, probabilistic (⅔, ½, ⅓, ¼, ⅕) rewards (e.g., €2 for certain or €5 with a 20% probability). The probability discounting for losses task applied the same procedure. Strong probability discounting (high *k* values) reflects risk aversion for gains, observed as preferring certain over probabilistic gains, whereas risk-seeking for losses manifests as preferring probabilistic over certain losses. Each of these three tasks comprised 30 trials, with offers from €0.30 to €10. In the mixed gambles task, participants received €10 before considering 40 gambles with a 50–50 chance of winning (€1–40) or losing (€5–20) each time. The tendency to reject gambling produces high λ values, indicating strong loss aversion.

In each trial, participants chose between two simultaneously presented options, which randomly appeared on the left and right sides of a computer screen in the laboratory. The chosen option was highlighted with a frame and used for Bayesian adaptive estimation to provide the next offer near individual indifference points. Slides encouraged realistic responses by instructing participants that monetary compensation for study participation would be adapted according to the outcomes of one randomly selected trial per task. To avoid confounding by attention, learning, and memory, the tasks included practice trials, had no time limit for making decisions, and displayed outcome contingencies without disclosing gambling outcomes. Choice consistency was evaluated by averaging mean-centered ß values across tasks, with high values representing consistent preferences across trials.

### Statistical analysis

Analyses were performed in the Statistics and AMOS modules of SPSS 28 (IBM, Armonk, NY, USA), assuming significance at *p* < 0.05. Latent growth curve modeling estimated inter-individual variability in intra-individual trajectories (described by latent intercept and slope) of alcohol involvement from age 18 to 24 and regressed this variability on choice preferences controlled for choice consistency at age 18. The models adjusted for measurement error and handled missing data on alcohol outcomes with full information maximum likelihood calculations (Burant, 2016).

The number of repeated measurements allowed for fitting linear and quadratic models for each alcohol outcome. The intercept to each repeated measurement was constrained to 0, allowing the means of repeated measures to be explained by the latent intercept and slopes. Regression weights between the latent intercept and repeated measurements were set to 1. Direct effects of the linear slope were weighted relative to the time point of each measurement. Loadings on the quadratic slope were the squares of the corresponding linear weights. Means and covariances of the latent intercept and slopes were freely estimated. Residual error terms of the latent intercept, slopes, and repeated measurements had 0-constrained means and unconstrained variances.

If the χ^2^-difference test did not indicate model improvement when adding a quadratic term, the linear model was selected and evaluated with the χ^2^ test, comparative fit index, Tucker–Lewis index, and root mean square error of approximation. The selected base models were checked for improper estimates, such as correlations exceeding 1 and negative variances. We added choice dimensions as mean-centered covariates to ease interpretation of their relations to trajectories of alcohol outcomes. Regression coefficients and covariances of the covariates were estimated freely. Final models were re-evaluated to ensure adequate fit and valid estimates. Significant predictions were plotted with model-implied trajectories of repeated measures at high (*M* + 1 SD), medium (*M*), and low (*M*–1 SD) values of single-choice dimensions, while holding the other covariates at their average (Biesanz et al., 2004). The area under the curve of average alcohol use was calculated at these three values to compare their cumulative consumption levels from age 18 to 24.

## Results

**Figure 1.**
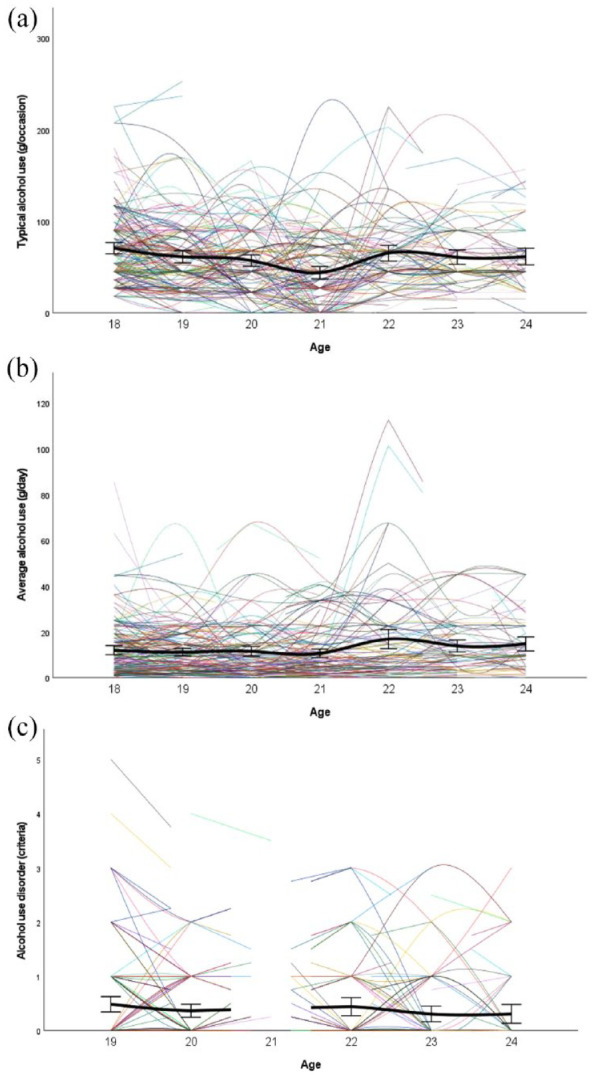
Alcohol involvement (a-c) from age 18 to 24. Colored lines represent individuals. Error bars are 95% confidence intervals of the group means.

Table 1 and Figure 1 present demographics of participants and their involvement with alcohol. Choice consistency was associated with choice preferences, which were not significantly inter-correlated except for loss aversion being associated with risk-seeking for losses and trend-level associated with risk aversion for gains (Table 2). Repeated measurements of alcohol outcomes were better represented by quadratic than linear models, except for Alcohol Use Disorder criteria (Table 3). Adequate fit was confirmed after incorporating covariates (Table 4).

**Table 1.** Demographics and alcohol involvement.

	Assessment at month						
	0	12	24	36	48	60	72
Participants	198	166	160	135	100	85	84
Education years	11.7 ± 0.7			14.2 ± 0.9			
Status							
Unemployed/homemaker	1.0	7.2	2.3	2.2			
Student	85.4	66.4	61.4	63.0			
Employee/trainee	13.6	26.4	36.4	34.8			
Age	18.4 ± 0.2	19.4 ± 0.2	20.4 ± 0.2	21.5 ± 0.2	22.4 ± 0.2	23.5 ± 0.2	24.6 ± 0.4
Of first whole alcoholic beverage	14.3 ± 1.4						
Of first alcohol binge (⩾ 5 drinks)	16.5 ± 0.8						
Of first subjective alcohol intoxication	15.7 ± 1.2						
Past-year alcohol use							
Typical (g/occasion)	70.5 ± 43.4	61.2 ± 42.4	57.0 ± 36.4	43.8 ± 39.0	65.1 ± 42.2	60.7 ± 35.8	61.4 ± 36.4
Average (g/day)	12.0 ± 14.0	11.1 ± 10.6	11.5 ± 12.4	10.6 ± 11.1	16.8 ± 20.4	13.8 ± 11.8	14.7 ± 12.3
Alcohol Use Disorder criteria		0.5 ± 0.9	0.4 ± 0.7		0.4 ± 0.8	0.3 ± 0.7	0.3 ± 0.7

Values are means ± standard deviations, except for participants (counts) and status (percentages). Past-year alcohol use was assessed with the Composite International Diagnostic Interview and its supplementary sheet displaying common and standard drinks (Kuitunen-Paul et al., 2017). Average consumption (g/day) was calculated by multiplying quantity (alcohol (g) converted from drinks (ml)) by frequency (abstinent, less than monthly, 1–3 days a month, 1–2 days a week, 3–4 days a week, almost daily; Kuitunen-Paul et al., 2017). Alcohol Use Disorder can be mild (2–3 criteria), moderate (4–5), or severe (6–11; American Psychiatric Association, 2013).

**Table 2.** Choice dimensions.

Choice dimensions	Delay discounting (log k)	Risk aversion for gains (log k)	Risk-seeking for losses (log k)	Loss aversion (log λ)
	−4.6 ± 2.2	−0.2 ± 1.0	−0.3 ± 1.2	0.3 ± 0.8
Risk aversion for gains (log k)	0.100 (0.174)			
Risk-seeking for losses (log k)	0.011 (0.876)	−0.009 (0.901)		
Loss aversion (log λ)	−0.024 (0.741)	0.141 (0.054)	−0.227 (0.002)*	
Choice consistency (log β)	−0.262 (<0.001)*	−0.186 (0.012)*	0.252 (<0.001)*	−0.211 (0.004)*

*N* = 198. Values are *M* ± SD or Pearson’s linear correlation coefficient *r* (0.1 = small, 0.3 = medium, 0.5 = large effect size; Cohen, 1988) with *p* values in parentheses (* = significant at 0.05).

**Table 3.** Comparison of base models.

Repeated measure	Linear model	Quadratic model	Model difference
Typical alcohol use	68.259 (23)	42.197 (19)	26.062 (4)*
Average alcohol use	59.483 (23)	34.596 (19)	24.887 (4)*
Alcohol Use Disorder criteria	12.923 (10)	10.779 (6)	2.144 (4)#

*N* = 198. Values are χ^2^ (df). If linear and quadratic models are significantly different from one another at 0.05 (*), the model with the lower χ^2^ is considered to fit the data better, otherwise (# = non-significant) it is the parsimonious (linear) model (Felt et al., 2017).

**Table 4.** Fit statistics of final models.

Repeated measure	χ2 (df), *p*	Comparative fit index	Tucker–Lewis index	RMSEA (90% CI)
Typical alcohol use	70.967 (39), 0.001	0.871	0.742	0.065 (0.040–0.088)
Average alcohol use	61.234 (39), 0.013	0.937	0.874	0.054 (0.025–0.079)
Alcohol Use Disorder criteria	26.944 (25), 0.359	0.981	0.959	0.020 (0.000–0.062)

*N* = 198. The χ^2^ test should be non-significant at 0.05 if the model adequately reflects the data. However, as larger sample sizes and more complex models are penalized, model evaluation should be based on a combination of tests (Felt et al., 2017; Burant, 2016). The comparative fit index, Tucker–Lewis index, and root mean square error of approximation (RMSEA) are not affected by sample size (Burant, 2016). Generally, comparative fit and Tucker–Lewis indices of 0.90 indicate acceptable and 0.95 good fit, while RMSEA values of 0.08 reflect acceptable and 0.05 good fit (Burant, 2016; Felt et al., 2017).

Table 5 lists the curve components of group trajectories for repeated measures (alcohol use and related problems) and the relation of covariates (choice preferences and consistency) to individual differences in trajectories. Intercept means (Table 5) are excellent reproductions of group means of recorded measures at their first assessment (Table 1). The linear factor in Table 5(c) describes the average change in the number of Alcohol Use Disorder criteria per year. In models for typical and average alcohol use (Table 5(a) and (b)), the linear component describes the initial rate of change, and the quadratic component describes how this rate changes on average per year. Significant negative linear and positive quadratic components of the group trajectory for drinking per typical occasion over 6 years describe an initial decrease followed by a later increase (Table 5(a)). Average daily drinking did not significantly change over time across men (Table 5(b)), whereas Alcohol Use Disorder criteria decreased at trend-level significance (Table 5(c)).

**Table 5.** Estimates of final models.

Group trajectory	(a) Typical alcohol use (g/occasion)		(b) Average alcohol use (g/day)		(c) Alcohol Use Disorder criteria	
	M ± SE	*p*	M ± SE	*p*	M ± SE	*p*
Intercept	69.856 ± 3.042	<0.001*	11.753 ± .914	<0.001*	0.443 ± .058	<0.001*
Linear	−8.680 ± 1.875	<0.001*	−0.275 ± 0.570	0.630	−0.030 ± .017	0.080
Quadratic	1.227 ± .312	<0.001*	0.099 ± 0.097	0.308	N/A	N/A
Delay discounting	β	*p*	β	*p*	β	*p*
Intercept	0.119	0.139	0.153	0.090	0.079	0.435
Linear	−0.160	0.158	−0.235	0.041*	−0.085	0.603
Quadratic	0.175	0.172	0.244	0.024*	N/A	N/A
Risk aversion for gains						
Intercept	0.086	0.278	0.128	0.151	−0.274	0.006*
Linear	−0.106	0.341	−0.262	0.020*	−0.011	0.943
Quadratic	0.086	0.495	0.204	0.055	N/A	N/A
Risk-seeking for losses						
Intercept	0.011	0.888	−0.101	0.268	−0.012	0.908
Linear	0.057	0.615	−0.009	0.938	−0.054	0.741
Quadratic	−0.070	0.585	0.056	0.609	N/A	N/A
Loss aversion						
Intercept	0.007	0.928	−0.075	0.410	−0.095	0.355
Linear	−0.232	0.044*	0.181	0.122	0.056	0.737
Quadratic	0.295	0.023*	−0.174	0.115	N/A	N/A
Choice consistency						
Intercept	−0.038	0.666	0.085	0.380	−0.072	0.512
Linear	−0.237	0.053	−0.164	0.186	0.007	0.968
Quadratic	0.284	0.040*	0.165	0.158	N/A	N/A

*N* = 198. Standardized partial regression effects (β) of covariates on group trajectories.

* Significant at 0.05.

**Figure 2.**
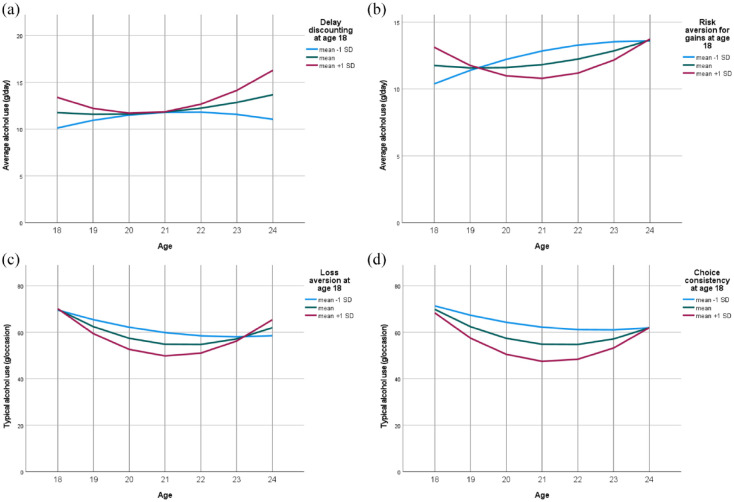
Alcohol outcomes from age 18 to 24 at three levels of choice behavior (a-d) at age 18. Partial regression effects are visualized as standard deviations from the model-implied mean trajectories of repeated measures when all other choice dimensions are at their centered mean.

The partial regression coefficients describe how a change in a covariate affects a change in curve components when all other covariates remain at their centered average (Table 5). Delay discounting at age 18 was trend-level associated with the intercept and significantly with the slope of average daily drinking over 6 years (Table 5(b)), linking high discounting to higher consumption in general and escalating consumption at the end of follow-up (Figure 2(a)). The area under the curve at high delay discounting (*M* + 1 SD) amounted to a cumulative alcohol intake of 28.3 kg in 6 years compared to 24.9 kg at low discounting (*M*–1 SD).

Risk aversion for gains significantly affected the intercept of Alcohol Use Disorder criteria (Table 5c) and the development of average daily drinking (Table 5(b)); risk-seeking at age 18 predicted meeting more Alcohol Use Disorder criteria in the following 6 years (Table 5(c)) and further increases in average consumption thereafter (Figure 2(b)). The area under the curve of average alcohol use amounted to a cumulative consumption of 27.5 kg in 6 years for low-risk aversion for gains (*M*–1 SD) compared to 25.7 kg for high-risk aversion (*M* + 1 SD). By contrast, risk-seeking for losses was not significantly related to alcohol outcomes (Table 5).

Loss aversion and choice consistency significantly shaped the development of drinking per typical occasion (Table 5(a)). Figure 2(c) and (d) indicates that high loss aversion and high choice consistency at age 18 predicted steeper decreases in typical drinking quantity until age 21, followed by rebounds toward baseline drinking by age 24.

## Discussion

This prospective observational study tested for predictive effects of delay discounting, risk aversion for gains, risk-seeking for losses, loss aversion, and choice consistency in healthy men at age 18 on alcohol use and related problems from age 18 to 24. The differential effects provide insights into cognitive-behavioral mechanisms by mapping specific choice dimensions on different trajectories of alcohol involvement.

### Decision-making predicts alcohol involvement

Medium and high expressions of loss aversion and choice consistency predicted short periods of alcohol consumption that exceeded 60 g per occasion. This is the threshold of the World Health Organization for heavy episodic drinking, which is associated with severe health and social harms (Thompson et al., 2014; World Health Organization, 2018). The risk of these harms may be increased profoundly by low loss aversion and low choice consistency at age 18, as either predicted heavy episodic drinking for almost the entire 6-year follow-up. Both high delay discounting and risk-seeking for gains at age 18 independently predicted a considerably higher cumulative alcohol use within the 6-year follow-up when average consumption remained stable at approximately 12.5 g per day. High delay discounting and risk-seeking for gains may also each increase the risk of alcohol-related harm, as the level of drinking that minimizes health loss is zero or very close to zero for several populations, particularly young adults (Bryazka et al., 2022).

Risk-seeking for gains additionally predicted meeting more Alcohol Use Disorder criteria in the following 6 years, whereas risk-seeking for losses was not significantly related to alcohol outcomes. The predictive effects collectively suggest that overvaluing immediate and probabilistic incentives, rather than underestimating harm, drives alcohol involvement in young men. These choice preferences were largely independent of each other but were correlated with choice consistency, which predicted heavy episodic drinking beyond these associations. Hazardous alcohol use may thus arise from various decision-making profiles, which could explain why a substantial number of people with Alcohol Use Disorder do not exhibit strong delay discounting (Green and Myerson, 2019). This also emphasizes that delay discounting cannot serve as a summary measure for general decision-making patterns (Bailey et al., 2021).

### Neurobiology of decision-making and related drinking

The predictive findings on alcohol outcomes may derive from underlying mechanisms involving striatal dopaminergic signaling because its manipulation with L-DOPA altered also delay discounting, risk aversion for gains, and loss aversion, but not risk-seeking for losses (Petzold et al., 2019a, 2019b). Another study in healthy adults targeted frontal dopamine and found a circuit mechanism for dopamine effects since delay discounting varied with tolcapone-induced changes in the left ventral putamen and the frontostriatal connectivity (Kayser et al., 2012). Drinking may thus escalate when decision-making processes in dopamine-innervated frontostriatal circuits are characterized by low executive control or high alcohol valuation (Camchong et al., 2014; Goschke, 2014; Sebold et al., 2022; Zucker, 2015). Chronic alcohol use reinforces this imbalance, thereby facilitating value-based impulsive and stimulus-driven habitual decisions that characterize dependent drinking (Camchong et al., 2014; Goschke, 2014; Sebold et al., 2022; Zucker, 2015).

### Targeting decision-making to reduce alcohol use

The imbalance between cognitive control and reward-seeking can also be addressed through psychological interventions (Rung and Madden, 2018; Verdejo-Garcia et al., 2019). Priming, framing, and episodic future thinking, among other techniques, reduced delay discounting in healthy volunteers (Rung and Madden, 2018). Framing contingencies and outcomes toward rewards, costs, or losses also swayed risk-seeking and loss aversion (Kahneman and Tversky, 1984). Episodic future thinking reduced drinking and delay discounting in adults with Alcohol Use Disorder (Athamneh et al., 2022). Moreover, contingency management and goal management training may be beneficial in substance use treatment by attenuating delay discounting and risk-seeking, respectively (Rung and Madden, 2018; Verdejo-Garcia et al., 2019). Sustained shifts in decision-making may be explained by strengthened choice consistency, given its inverse correlation with delay discounting in our study. Considering various choice dimensions can also inform policy interventions (e.g., framing warning messages). Targeting decision-making seems particularly promising, considering that high delay discounting, risk-taking, and alcohol use are all implicated in myriad injuries and diseases (Bickel et al., 2012, 2019; Bryazka et al., 2022; World Health Organization, 2018).

### Limitations

We assessed the predictive value of important but certainly not all domains of value-based decision-making; for example, people with Alcohol Use Disorder also exhibited strong discounting of delayed losses (Bailey et al., 2018; Gerst et al., 2017). Heavy episodic drinking occurred frequently over 6 years, but average consumption remained substantially below the worldwide average of 32.8 g per day in people who drink alcohol (World Health Organization, 2018). Moreover, the spectrum of alcohol-related problems during follow-up was insufficient to clarify the contributory dimensions of decision-making in all severities of Alcohol Use Disorder. Value-based decision-making may however not be specific to substance-related problems, as delay discounting explained variance in substance use but not in related problems when controlling for substance use (Hildebrandt et al., 2021).

The lack of follow-up data after age 24 precludes claims about predictive effects on alcohol outcomes later in life. Some predictions may also not generalize to other demographics (e.g., women) and contexts (e.g., education) that affect choice preferences, drinking patterns, or their associations. Our data were collected in Germany, which has a minimum age of 16 for purchasing beer/wine and 18 years for purchasing spirits (World Health Organization, 2018). The most common minimum age for alcohol purchase is 18, followed by 21 and 16 years (World Health Organization, 2018). Countries without a minimum age for alcohol purchase tend to be low-income or lower-middle-income (World Health Organization, 2018). Poverty is linked to hazardous alcohol use (World Health Organization, 2018) and strong delay discounting, but may trigger risk aversion for gains (Green and Myerson, 2019).

## Conclusions

High delay discounting, risk-seeking for gains, low loss aversion, and low choice consistency may each and thus more so combined increase the risk of hazardous drinking among young men. In contrast, risk-seeking for losses may hold no predictive value. The differential relationships between these choice dimensions and the development of average alcohol use, heavy episodic drinking, and associated problems describe distinct cognitive-behavioral patterns. Future research will determine whether these patterns can advance risk stratification and guide the deployment of interventions targeted at decision-making to reduce alcohol-related harm.
